# Understanding the electronic structure of Y_2_Ti_2_O_5_S_2_ for green hydrogen production: a hybrid-DFT and GW study†

**DOI:** 10.1039/d3ta02801a

**Published:** 2023-07-20

**Authors:** Katarina Brlec, Christopher N. Savory, David O. Scanlon

**Affiliations:** a Department of Chemistry and Thomas Young Centre, University College London London UK d.scanlon@ucl.ac.uk

## Abstract

Utilising photocatalytic water splitting to produce green hydrogen is the key to reducing the carbon footprint of this crucial chemical feedstock. In this study, density functional theory (DFT) is employed to gain insights into the photocatalytic performance of an up-and-coming photocatalyst Y_2_Ti_2_O_5_S_2_ from first principles. Eleven non-polar clean surfaces are evaluated at the generalised gradient approximation level to obtain a plate-like Wulff shape that agrees well with the experimental data. The (001), (101) and (211) surfaces are considered further at hybrid-DFT level to determine their band alignments with respect to vacuum. The large band offset between the basal (001) and side (101) and (211) surfaces confirms experimentally observed spatial separation of hydrogen and oxygen evolution facets. Furthermore, relevant optoelectronic bulk properties were established using a combination of hybrid-DFT and many-body perturbation theory. The optical absorption of Y_2_Ti_2_O_5_S_2_ weakly onsets due to dipole–forbidden transitions, and hybrid Wannier–Mott/Frenkel excitonic behaviour is predicted to occur due to the two-dimensional electronic structure, with an exciton binding energy of 0.4 eV.

## Abstract 2

Combined hybDFT and GW study reveals surface properties and optoelectronic behaviour of Y_2_Ti_2_O_5_S_2_ for green hydrogen production.

## Graphical abstract

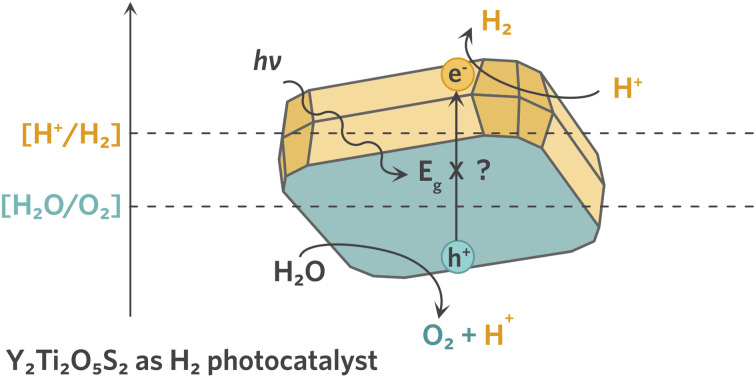

Hydrogen is often advertised as a green and clean fuel of the future, but it already is an important chemical feedstock. Worldwide, 96% of hydrogen is used in the chemical industry, mostly in production of ammonia fertilisers and hydrocarbon refining.^1^ However, despite its status as the most abundant substance in the universe, it is very scarce in its most useful H_2_ diatomic form on Earth, where it exists primarily in water, hydrocarbons and other organic matter.^2,3^ Hydrocarbons have been traditionally used in production of hydrogen, with coal gasification and steam methane reforming among the most popular methods. Both are highly energy-intensive and emit large quantities of CO_*x*_ gases (even when accounting for the proposed use of carbon capture), which is clearly unsustainable long-term.^4,5^

A clear alternative to hydrocarbon reforming lies in the other major source of hydrogen – water. In 1972, Honda and Fujishima discovered that hydrogen can be produced by water electrolysis using ultraviolet (UV) light shone on a TiO_2_ electrode, popularising the idea of photocatalysis.^6^ TiO_2_ has since become one of the most studied photocatalytic materials due to its high photocorrosive stability, ease of manufacture and non-toxicity.^7–9^ However, because of its wide band gap (3.2 eV) titania can only absorb UV light, which only comprises less than 5% of the entire incident light on Earth.^10^

To strike the balance between absorption of a wide light spectrum and satisfying the thermodynamic and kinetic demands of water splitting, 1.8–2.2 eV has been identified as the ideal band gap range for photocatalysis.^11^ While exceptionally stable in water, metal oxides suffer from low-lying O 2p states that push the valence band maximum down in energy, opening the band gap too wide. Sulphides, on the other hand, contain S 3p states that are higher in energy which close the band gap substantially without altering the conduction band edge. Unfortunately, sulphides tend to self-oxidise and photocorrode in a water medium, which makes them unsuitable as photocatalysts.^12^ However, the photocorrosion stability of oxides and favourable electronic structure of sulphides can be combined in mixed-anion oxysulphides.

The family of cation-deficient Ruddlesden-Popper Ln_2_Ti_2_O_5_S_2_ (Ln = Y, Nd, Sm, Gd, Tb, Dy, Ho and Er) compounds exhibit this behaviour with measured optical band gaps in the 1.8–2.2 eV.^13,14^ Sm_2_Ti_2_O_5_S_2_, Gd_2_Ti_2_O_5_S_2_ and Tb_2_Ti_2_O_5_S_2_ have been identified as promising photocatalysts for water splitting.^15^ All three compounds exhibit rates of H_2_ and O_2_ evolution of about 20 μmol h^−1^ under visible light in the presence of sacrificial electron donors and acceptors.^15^ Y_2_Ti_2_O_5_S_2_ demonstrated simultaneous stoichiometric production of H_2_ and O_2_ when loaded with Rh/Cr_2_O_3_ and IrO_2_ as hydrogen and oxygen evolution co-catalysts, respectively, achieving rates of about 2.5 μmol h^−1^ and 1.25 μmol h^−1^, resulting in an apparent quantum yield (AQY) of 2.3% under UV light.^16^ Higher evolution rates of were 80 μmol h^−1^ (H_2_) and 40 μmol h^−1^ (O_2_) achieved when the co-catalysts were loaded and tested as separate half-reactions, so Y_2_Ti_2_O_5_S_2_ performs better compared to its lanthanide counterparts. More recently an improved AQY for oxygen evolution of 6.3% under 420 nm light irradiation was reported with Co_3_O_4_ co-catalyst loading.^17^

With an experimental band gap of 1.9 eV, predicted suitable band alignment for water redox and calculated low hole and electron effective masses of 0.5 *m*_e_ in the in-plane direction, Y_2_Ti_2_O_5_S_2_ indeed has all the indicators of a promising earth-abundant photocatalyst.^16,18^ While several computational studies have focused on the properties of the bulk Y_2_Ti_2_O_5_S_2_, this study aims to unveil the surface properties using state-of-the-art computational methodology.^18,19^ In particular, this study explores surface behaviour and energetics during relaxations, and how that relates to the band edge positions of the surfaces. Additionally many-body perturbation theory calculations are performed to calculate bulk optoelectronic properties, including the exciton binding energy, to support our hybrid DFT calculations and explore the optical behaviour of the system.

## 1 Computational methodology

**Fig. 1:**
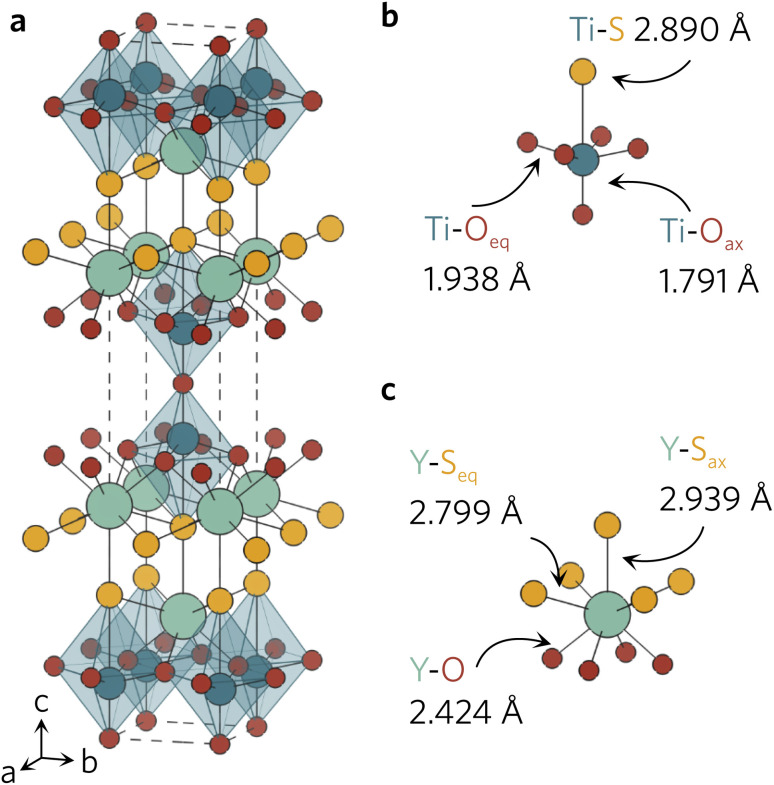
(a) Conventional unit cell of Y_2_Ti_2_O_5_S_2_ and local bonding environments of the TiO_5_S octahedra (b) and YO_4_S_5_ motifs (c) as calculated with HSE06. The atom colours are as follows: Y = light green, Ti = dark blue, S = yellow, O = red. The unit cell is represented with a dashed line. Plotted with VESTA.^28^

All density functional theory (DFT) calculations were performed with the Vienna *Ab initio* Simulation Package (VASP), using the recommended projector-augmented wave (PAW) potentials to simulate the core and valence electrons.^20–23^ The same converged plane wave energy cutoff (480 eV) and *k*-point meshes (5 × 5 × 5 for primitive, 5 × 5 × 1 for conventional) were used as in a previous study.^18^ The crystal structure (Fig. 1a) obtained from Materials Project was relaxed into the ground state using the generalised-gradient approximation (GGA) Perdew–Burke–Ernzerhof functional revised for solids (PBEsol) and the hybrid-DFT Heyd–Scuseria–Ernzerhof (HSE06) functional.^24–26^ The conventional unit cell was relaxed with no constraints on unit cell shape and volume, until the maximum force on any atom did not exceed 0.01 eV Å^−1^, using a higher energy cutoff (620 eV) to account for Pulay stress.^27^

Because VASP cannot restrain the periodicity into just two dimensions, the slab model was employed herein to study the surface properties. The surfaxe package was used to cleave the slabs, calculate the parameters used in convergence testing and perform the relevant analyses.^29^ All non-polar, symmetric slabs up to a maximum Miller index of two were cleaved from the relaxed conventional unit cell.

To ensure the slabs were an accurate representation of the bulk and surfaces, extensive convergence testing to determine the minimal slab size required was conducted. For each of the Miller indices, the positions of atoms in the surface slabs with slab thicknesses ranging 10 to 50 Å were relaxed with PBEsol, while the unit cell shape and size was kept consistent. The average (arithmetic mean) bond distance change with respect to the atom's position in *c*-direction was used to ensure the bulk-like region was sufficiently large. If the average bond length close to the centre of the slab was close to the bond length of the same character in the bulk, then the slab was deemed to be sufficiently thick. The surface energy was also converged against slab thickness, where a criterion of 0.02 J m^−2^ was used to check convergence. Lastly, vacuum size was chosen to be 20 Å as this was the minimum thickness at which the planar potential was flat across all Miller indices.

For calculation of the band alignment of surfaces, the slabs were cleaved from the HSE06-relaxed bulk conventional unit cell. The atomic positions were then relaxed with HSE06 using the same force criterion as before, with constraints on unit cell shape and volume. This was done as hybrid functionals offer a better description of the electronic structure compared to GGA functionals, so the band alignment presented is more accurate.

Finally, the bulk effective charge carrier masses, dielectric constant and optical absorption spectrum discussed here were calculated as a part of a previous study.^18^

To assess the character of the exciton behaviour in Y_2_Ti_2_O_5_S_2_, many-body quasiparticle electronic structures and optical spectra were calculated within the Questaal package^30^ through the quasiparticle self-consistent *GW* (QS*GW*) method.^31^ Questaal utilises a full-potential linear muffin-tin orbital basis: augmentation sphere radii (in atomic units) of 2.80, 2.10, 2.49 and 1.52 were used for Y, Ti, S and O atoms respectively, with automatically generated interstitial smooth Hankel functions and an *l*-cutoff of 4 used for all atoms. Local orbitals were necessary to include the Ti 3p and Y 4p within the valence electrons. A GW *k*-mesh of 5 × 5 × 5 and interstitial *G*-vector cutoff of 
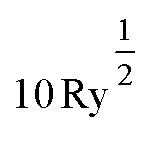
 were found to converge the quasiparticle gap to within 10 meV, with a 7 × 7 × 7 *k*-mesh used to obtain the initial PBE exchange–correlation potential. QS*GW*, which tends to demonstrate excellent agreement with experimental band gaps for s–p dominated semiconductors, overestimates band gaps systematically due to the usage of the Random Phase Approximation (RPA) – this can be improved by including ladder diagrams, which can describe the additional contribution by explicit two-particle electron–hole interaction to the screened Coulomb *W*;^32^ these can be obtained *via* the Bethe–Salpeter equation (BSE), and their additional to QS*GW* has lead to exceptional accuracy with respect to experimental band gaps in previous cases.^33–35^ The inclusion of these effects within a self-consistent calculation of the self-energy is here denoted QS*GŴ*, and within the explicit solution of optical transitions, to obtain excitonic features, QS*GŴ* + BSE (without, QS*GŴ* + RPA). Both QS*GW* and subsequent QS*GŴ* self-consistent cycles were iterated until the root-mean-square change in the self-energy was below 1 × 10^−5^. In the solution of the BSE, 8 valence and 8 conduction bands were used, with a *k*-mesh of 7 × 7 × 7 used for the optical spectrum, which ensured the dielectric constant (*q* → 0) was converged to within 0.01.

## 2 Results and discussion

The relaxed conventional unit cell can be seen in Fig. 1, together with the TiO_5_S and YO_4_S_5_ units. Compared to the experimental structure by Hyett *et al.*^36^ (*I*4/*mmm*, *a* = 3.770 Å, *c* = 22.806 Å), the volume of the unit cell decreased by 1.3% (PBEsol; *I*4/*mmm*, *a* = 3.755 Å, *c* = 22.682 Å) and 0.5% (HSE06; *I*4/*mmm*, *a* = 3.756 Å, *c* = 22.871 Å). The calculated lattice parameters clearly agree well with the experimental data, with slight differences between the two functionals in the out-of-plane *c* parameter.

The larger volume decrease in the PBEsol-relaxed structure can be predominantly attributed to the shortening of Ti–S (−0.021 Å) and Y–S_ax_ (−0.048 Å), whereas the bond distances were elongated by 0.022 Å and 0.017 Å in the HSE06 structure (see Table 1). For the interested reader, a more detailed description of the changes in bond distances in Y_2_Ti_2_O_5_S_2_ conventional unit cell during the relaxations with different functionals can be found in our previous work.^18^

**Table 1:** Y_2_Ti_2_O_5_S_2_ bond distances in Å as calculated with PBEsol and HSE06, compared with experimental values from powder X-ray diffraction by Hyett *et al.*^36^ (Exp.) and Materials Project data (MP, accessed April 2020).^37^ The percentage difference between calculated and experimental bond distances has been calculated relative to the crystal structure reported by Hyett *et al.*^36^

Bond	PBEsol	HSE06	Exp.	PBE (MP)
Ti–O_ax_	1.800	1.791	1.794	1.816
	(+0.3%)	(−0.2%)		
Ti–O_eq_	1.936	1.938	1.943	1.959
	(−0.4%)	(−0.3%)		
Ti–S	2.868	2.890	2.872	2.919
	(−0.1%)	(+0.6%)		
Y–O	2.412	2.424	2.433	2.455
	(−0.9%)	(−0.4%)		
Y–S_ax_	2.885	2.939	2.933	2.954
	(−1.6%)	(+0.2%)		
Y–S_eq_	2.797	2.799	2.802	2.830
	(−0.2%)	(−0.1%)		

### 2.1 Surface construction and relaxation

All surface slabs studied were non-polar and contained inversion symmetry. The latter requirement is directly related to the slab model employed as it assumes the two surfaces created are equivalent. The importance of non-polarity is twofold; (1) polar surfaces are typically higher in energy, so less likely to spontaneously form and (2) charged surfaces are difficult to accurately simulate due to the additional dipole corrections required in VASP. When cleaving the surfaces, no restrictions were placed on the bonds broken as none of the bonds in the compound were identified to be strong covalent bonds. In total, one non-polar symmetric termination was identified for each of the eleven Miller indices under investigation. The constructed slabs have bulk-like surfaces, left in a high energetic state due to the large number of dangling bonds produced by the cleavage. To account for this, all atoms were relaxed to find a more favourable, lower energy configuration. All bulk-like and relaxed slabs can be found in the ESI† data repository.

**Fig. 2:**
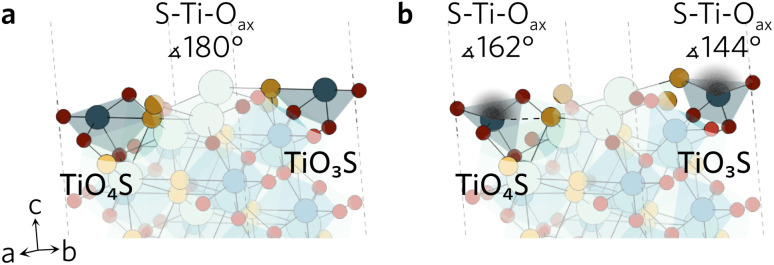
The (211) (a) unrelaxed and (b) relaxed surfaces highlighting the changes in S–Ti–O_ax_ bond angles of the two different TiO_*x*_S.

While the surface slabs created differ greatly in size and complexity, some general trends in relaxations were observed regardless. Displacements of atoms greater than 0.2 Å were contained within the topmost two layers in all slabs. Many surfaces were cleaved so that two Ti–O_eq_ (equatorial O) bonds were cut and the Ti was left in a TiO_3_S unit. The octahedral shape of TiO_5_S cannot be maintained in such a composition so the axial O (O_ax_) and S were pushed up towards the vacuum to form a more tetrahedron-like shape. Typically S–Ti–O_ax_ bond angle decreased to about 140° from 180° and S displaced more from its original position than O_ax_. Similar behaviour was seen when only one Ti–O_eq_ bond was cleaved, but to a much lesser extent so that the S–Ti–O_ax_ bond angle only decreased by 20°. Both TiO_3_S and TiO_4_S cases are highlighted in Fig. 2 featuring the (211) surface.

**Fig. 3:**
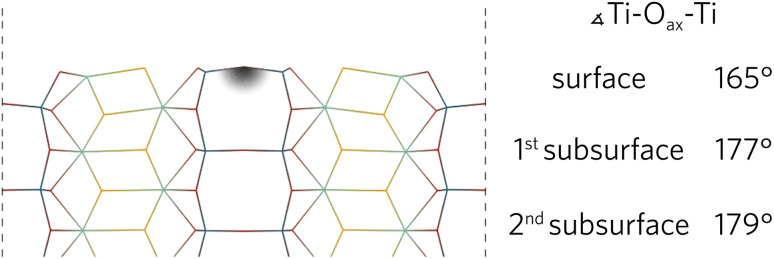
The (100) relaxed surface highlighting the layer-by-layer changes of the Ti–O_ax_–Ti bond angles parallel to the surface.

During the relaxation of slabs where Ti–O_ax_–Ti bonds were roughly parallel with the surface, the O_ax_ in the surface layer was pushed up towards the vacuum. As can be seen in Fig. 3 for the (100) slab, the Ti–O_ax_–Ti bond angle typically decreased by about 15°. The effects of the relaxation on this bond tapered off quickly as the first subsurface Ti–O_ax_–Ti bond angle decreased by less than 5° in all slabs, while the second subsurface angle is closer to 180°.

The average bond distance was calculated for all four different bonds in the slabs using surfaxe. In all slabs, the bonds on the surface were significantly shorter compared to the bulk-like bond lengths. Select surface bond distance data can be found ESI Tables S1–S4,† while the bulk bond lengths can be seen in Table 1. The bond distances in the subsurface layer was typically slightly longer, which is in line with the expected pattern of relaxations normal to the surface. The surface unit layer is expected to contract when no reconstructions are observed to account for the extra energy from the dangling bonds on the surface, while the first subsurface layer expands to counteract that. A slight contraction is sometimes observed for the second subsurface layer, however the bulk-like bonding is regained in the third subsurface layer in all slabs. Where unit layers are composed of more than four atomic layers, the bulk-like behaviour is observed within the first subsurface layer.

The slab thicknesses at which the average bond distances converged to bulk-like can be found in Table 2. The convergence of slab thicknesses with respect to average bond lengths is limited by the convergence of bonds lengths in bonds that are most perpendicular to the surface. This is directly related to the aforementioned contraction and expansion of the unit layers due to excess energy on the surface. In all slabs the slowest convergence was seen for either the Ti–S or Y–S bonds, possibly because of longer bonding distances, indicating weaker bonding compared to M−O bonds. When TiO_3_S segments were exposed on the surface, S always displaced more than O_ax_, which had a bigger effect on the subsurface layers. The Ti–S bond lengths may seem unusually long in the bulk Y_2_Ti_2_O_5_S_2_ (2.87 Å) when compared to materials like TiS_2_ (2.43 Å) or TiS (2.47 Å), but they actually compare well with Ti–S bond lengths in La_5_Ti_2_AgS_5_O_7_ (2.90 Å) and La_5_Ti_2_CuS_5_O_7_ (2.80 Å) where a similar TiO_5_S octahedral environment is observed.^38^

**Table 2:** Converged slab thicknesses for each of the Miller indices studied, in Å, number of unit layers and number of atoms

Miller index	Slab thickness (Å)	Unit layers	Atoms
(001)	43	2	44
(100)	28	8	172
(101)	32	9	99
(102)	34	9	198
(111)	34	12	264
(112)	32	12	132
(201)	44	11	484
(210)	21	6	264
(211)	40	9	198
(212)	26	7	308
(221)	40	16	704

In a number of slabs the relaxations at the surface caused a slight change in coordination to some atoms in the surface and first subsurface layers. The contraction of the surface TiO_3_S into a tetrahedral-like shape in (111), (112), (210) and (221) resulted in the S atom displacing by over 1.5 Å. As the S atom displaced outwards, this led to an over 1 Å increase in the bond distance between S and Y in the subsurface layer, so that bond is effectively broken. The same process occurs on the (211) surface, however the description of the surface relaxation is complicated by the presence of a TiO_4_S unit on the surface. During the relaxation, the Ti–S bond distance in the TiO_4_S increases to 3.22 Å (shown with a dashed line in Fig. 2b), creating a TiO_4_ and keeping the subsurface layer coordination intact.

Perhaps the most striking differences in the surface and subsurface layers were seen in the (212) slab. The six cleaved Ti–O bonds led to many changes in coordination, driven by the relaxations of TiO_3_S and two Ti_4_OS. All three units relaxed identically to the counterparts on the (111), (112) and (211) surfaces. The last under-coordinated Ti on surface was left in a TiO_3_ unit with missing Ti–S and two Ti–O_eq_ bonds. During the relaxation the O_ax_–Ti–O_eq_ bond angle in the TiO_3_ increased from 104° to 119°, pushing the O_eq_ outwards. This relaxation breaks the Ti–O_eq_ bond in the subsurface layer.

**Fig. 4:**
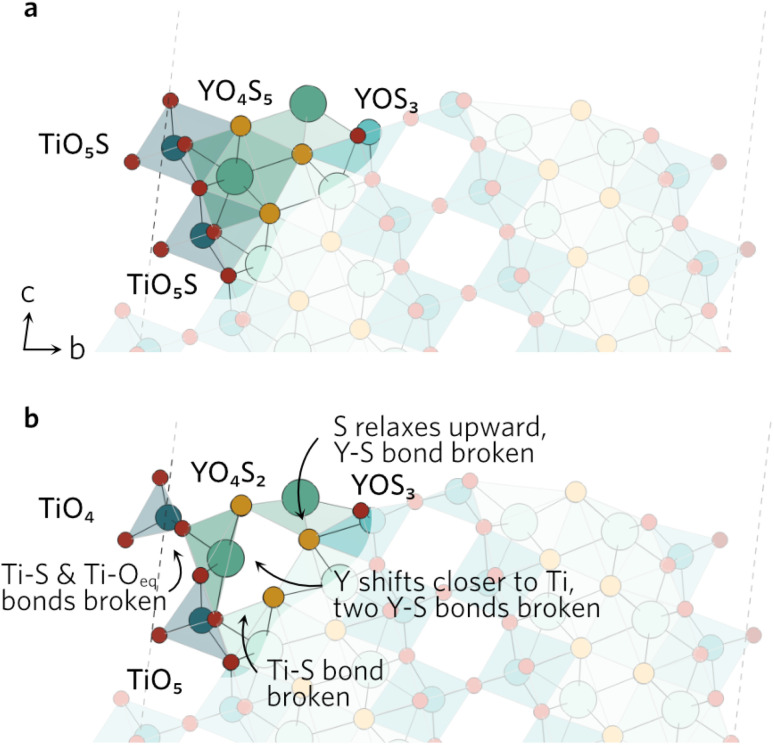
The (102) (a) unrelaxed and (b) relaxed surfaces.

In the (102) slab (see Fig. 4), the under-coordinated YOS_3_ on the surface flattened out during the relaxation to decrease the bond angle from 160° to 128°. This increases the Y–S bond length by 0.55 Å to 3.44 Å in the neighbouring fully-coordinated YO_4_S_5_, effectively breaking that bond. The increase also comes as a result of the Y atom of the YO_4_S_5_ unit relaxing towards the Ti, which breaks two Y–S bonds to S in the subsurface layer. Lastly, the largest displacement by any atom in the (102) slab was seen by the surface Ti in the TiO_5_S with Ti–S bond pointing outwards. The Ti atom shifts by over 0.8 Å towards the surface, breaking one of the Ti–O_eq_ bonds as bond distance increases to 2.67 Å. The O_ax_–Ti–O_eq_ angles in the newly formed TiO_4_ decrease to resemble an elongated tetrahedral shape.

**Fig. 5:**
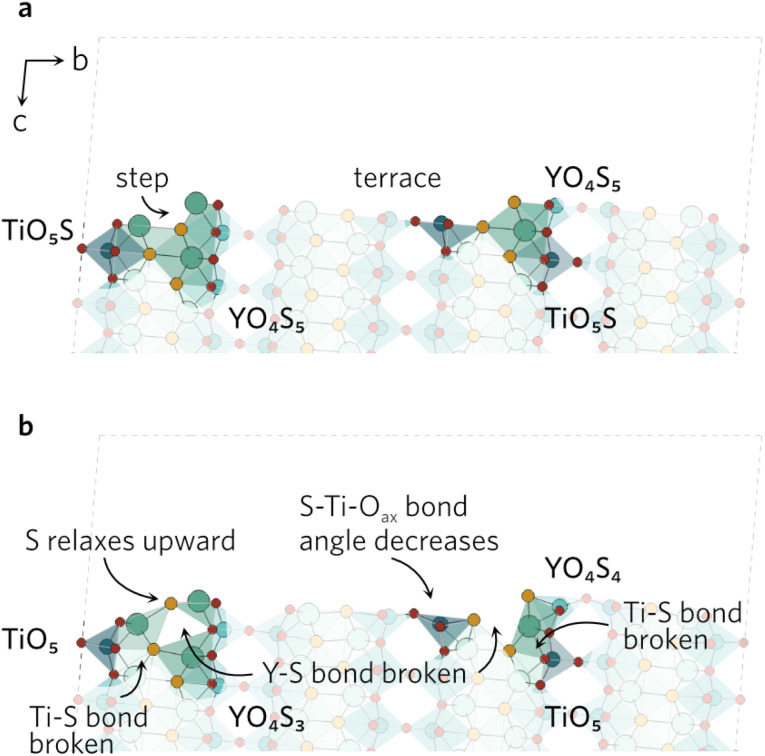
The (201) (a) unrelaxed and (b) relaxed surfaces.

The clearly defined steps (see Fig. 5) on the (201) surface morph during the relaxation. The O_ax_–Ti–S bond angles of all four neighbouring ‘terrace’ TiO_4_S units decrease during the relaxation, which causes a discontinuity in the step. As the bond angles close up, the Y–S bond distance on the step increases to 3.84 Å, effectively rendering the bond broken. The change in the other step is governed by the relaxation of the under-coordinated YO_3_S_2_, where S relaxes upwards to flatten out to a square-pyramidal-like shape. The knock-on effects from this relaxation mean the bonding in the first subsurface layer of nearby atoms is disrupted, resulting in broken Ti–S and Y–S bonds.

Slabs relaxed with hybrid HSE06 functional experienced the same relaxations as with PBEsol, so a repeat description is not necessary.

### 2.2 Surface energies and Wulff construction

Surface energies (*γ*) of relaxed slabs were calculated using the Fiorentini and Methfessel (FM) method, where the bulk energy (*E*_bulk,FM_) is first derived from a line of best fit for a number of slab DFT energies (*E*_slab_) for slabs with thickness *M*:

1 *E*_slab_(*M*) = 2*γ* + *ME*_bulk,FM_.

The surface energy is then calculated from the standard equation:2
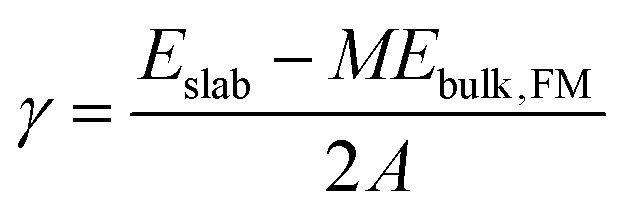
where *A* is the area of the surface and the factor 2 reflects the two equivalent surfaces that are created. Compared to calculating the surface energy directly from eqn (2) where the bulk DFT energy must be explicitly stated, the FM method removes the variable from the equation by calculating bulk energy implicitly. The FM method thus side-steps the issue of different reciprocal space sampling between bulk and surface calculations, which is the reason for diverging surface energies with increasing slab thickness. The slab DFT energies for this calculation were obtained from PBEsol single shot calculations using Gaussian *k*-point smearing using the relaxed slabs. For the (221) Miller index, the 50 Å slab contained 878 atoms which makes the relaxation computationally too expensive to justify even at the GGA level, so only three data points are used in the FM fit. For completeness, the cleavage energies of unrelaxed slabs were also calculated using the same method. Cleavage and surface energies of all slabs can be found in Table 3.

**Table 3:** Cleavage and surface energies calculated with PBEsol using the Fiorentini–Methfessel (FM) method in J m^−2^

Miller index	Cleavage energy	Surface energy
(001)	0.41	0.39
(100)	1.48	1.03
(101)	1.43	0.99
(102)	2.83	1.99
(111)	2.91	1.73
(112)	2.77	1.61
(201)	1.73	1.14
(210)	2.09	1.17
(211)	2.05	1.15
(212)	2.36	1.35
(221)	2.93	1.75

Lower Miller index surfaces have lower cleavage and surface energies compared with higher order surfaces, likely a result of fewer bonds broken in the formation of the lower index surfaces. The (100) and (101) surfaces see modest 0.4 J m^−2^ reductions in energy upon relaxations, while the surface energy of (001) remains almost unchanged with a small 0.02 J m^−2^ difference in energy. The (101) surface is slightly lower in energy than (100) even though the opposite trend would be typically expected. This can be explained by considering the bonds broken during the cleavage as two Ti–O_eq_ and four Y–S bonds were broken on the (100) surface, while on the (101) only one Ti–O_eq_, two Y–S and one Y–O bonds were broken. The 0.05 J m^−2^ difference in the cleavage energies between the two is maintained after the relaxation.

With the exception of the (201) surface, all higher order surfaces had cleavage energy exceed 2 J m^−2^. However, once they were relaxed, the surface energies were about 1 J m^−2^ lower than in energy, indicating a large relaxation had occurred. Referring back to the discussion earlier in Section 2.1, all the surfaces that saw a drastic reduction in excess energy were the ones where the surface TiO_3_S units were able to contract into a more tetrahedral shape.

**Fig. 6:**
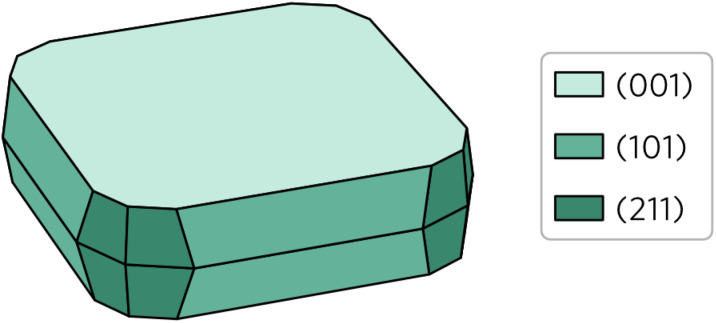
Wulff construction of Y_2_Ti_2_O_5_S_2_. The surfaces are colour coded so that the lighter shades represent lower surface energies and *vice versa*.

Wulff construction of Y_2_Ti_2_O_5_S_2_ equilibrium form under thermodynamic limits was created based on the surface energies and the bulk lattice. The central idea behind the prediction of particle shape based on Wulff constructions is that lower energy surfaces will be more likely to form than higher energy surfaces. As shown in Fig. 6, this is indeed the case for Y_2_Ti_2_O_5_S_2_ Wulff shape. Unsurprisingly, the (001) facet occupies by far the largest area, covering over 55% of the total area. The other 45% is composed of the (101) facet covering 30% and the (211) facet covering 15% of the area. The (100) surface does not feature on the Wulff shape despite its energy being lower than that of the (211) surface.

The calculated Wulff construction is in excellent agreement with previously reported experimental particle shapes. Pan *et al.*^39^ described the synthesised particles as ‘plate-like’, with a clear basal (001) plane, which is in line with what we observe here. Considering they also report the particles like to form secondary clusters combining several primary particles, a visual comparison of the Wulff shape with the scanning electron microscopy images from the studies by Pan *et al.*^39^ and Wang *et al.*^16^ show good agreement between the two.

Nevertheless, the calculation of surface energies lacks the relation to the real-world conditions of photocatalytic water splitting, where the material would be submerged in water and exposed to air. While the surface eergy equation (eqn (2)) can be extended to include terms to account for solvation and adsorption of water molecules on the surface, the computational cost associated with the calculations of these terms outweighs their usefulness in this study. Still, some general trends and conclusions may be drawn from the literature. An increase in hydration generally leads to a decrease in surface energy, which means the higher energy surfaces would be more likely to experience a stabilising effect.^40,41^ The decrease in surface energies may also lead to a smaller range of surface energies, so the Wulff shape may become more isotropic.

### 2.3 Band alignment

Band alignments of the Y_2_Ti_2_O_5_S_2_ surfaces that appeared on the Wulff shape were calculated using the core level alignment approach.^42,43^ The O_eq_ with two Ti and two Y atoms as nearest neighbours was chosen as the reference ‘core level’ atom to relate and compare the bulk and slab energies. Surfaxe was used to ensure the core level energy came from the O_eq_ closest to the mid-point of the slab. A more detailed description of calculation of the method used can be found in our previous work.^18,38^

**Fig. 7:**
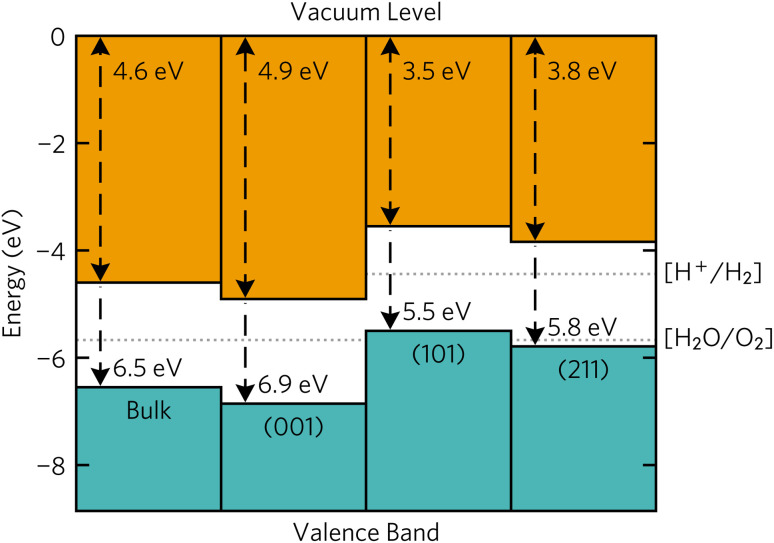
Calculated band alignments of bulk Y_2_Ti_2_O_5_S_2_ and the (001), (101) and (211) Y_2_Ti_2_O_5_S_2_ surfaces. Plotted with bapt.^44^

The surface band alignments are shown in Fig. 7 together with the bulk alignment. The (001) surface exhibits band alignment suitable for thermodynamic and kinetic photocatalysis of the oxygen half reaction with a large 1.1 eV overpotential at the valence band edge. Contrarily, the (101) and (211) surfaces are aligned for thermodynamic and kinetic photocatalysis of the hydrogen half reaction due to the 0.74 eV overpotential. The stark difference in band edge positions between surfaces can be ascribed to 1 eV larger surface dipole between the macroscopic planar potential and vacuum potential in the (001) surface (10.7 eV) compared to the (101) and (211) surfaces (9.5 eV). However, the macroscopic planar potential and core energies energy differences between the three surfaces are within 0.3 eV of each other, so the reason why the band edges are different must stem from the vacuum potential of the slabs.

Similar variation in band offsets between different facts was observed in many other systems. The calculated ionisation potentials between different facets of SnS varied by as much as 0.9 eV from the lowest-energy (100) facet.^45^ In In_2_O_3_, a valence band offset of 0.7 eV was observed both experimentally from X-ray photoelectron spectroscopy (XPS) and computationally from hybrid-DFT for the non-polar (111) and polar (100) surfaces.^46,47^ The same computational study also showed a 0.5 eV offset between the non-polar (111) and (110) facets.^47^ The fluctuations were also seen in quinary La_5_Ti_2_MS_5_O_7_ (M = Ag, Cu) where the conduction bands of the (100), (101) and (11̄2) facets were offset from the other facets' conduction band edges by almost 2 eV.^38^

Combining the band edge positions with Wulff construction, it can be seen that the facets that favour each of the half-reactions are almost equally split. The spatial separation of the hydrogen and oxygen evolution facets has been observed experimentally. The efficiency of water splitting could be potentially improved if the deposition of reduction and oxidation co-catalysts was separated between the basal (001) facets and the side (101) and (211) facets.

Of course, as with the surface energies, the ionisation potentials and electron affinities are calculated under the assumption that the surfaces form an interface with vacuum. The presence of a surface/water interface in a real aqueous solution will alter the position of band edges, reflecting the intrinsic nature of the semiconductor.^48^ An upward shift of the bands due to the electron transfer between water and the material is expected for natively n-type semiconductors. Based on literature experimental results and the bulk band alignment favouring n-type behaviour based on doping rules, Y_2_Ti_2_O_5_S_2_ is expected to be intrinsically n-type, so an upward shift of band edges may be expected. This is in line with experimental observations of photocatalytic activity, as poorer O_2_ evolution rates are expected based on the lower kinetic overpotential available on the (001) surface.

Accurately quantifying the band offsets from solvation of water on Y_2_Ti_2_O_5_S_2_ is beyond the scope of this study as it would be prohibitively expensive to do at a hybrid-DFT level. Nevertheless, the literature offers some suggestions on the quantitative effects of the solvation. Stevanović *et al.*^49^ showed that when pH equals the point of zero charge, the effect of the aqueous solution can be approximated by a 0.5 eV upward shift of band edges for metal oxides.

### 2.4 Identifying surface active sites

It is widely recognised that surface photocatalytic activity can be enhanced by modifying its surfaces using defect engineering. For example, in In_2_O_3_ a 15-fold improvement in visible light photocurrent was noted when the oxygen vacancies were introduced on the surface of the porous sheets.^50^ Similarly, about 3% (atom) concentration of oxygen vacancies in SrTiO_3_ improved the hydrogen evolution rates under UV-visible light by 2.3-fold compared to the unmodified SrTiO_3_.^51^

A full intrinsic point defects study for the Y_2_Ti_2_O_5_S_2_ surfaces is well beyond the scope of this study, however Madelung potential can be used *in lieu* as a coarse prediction tool for defect behaviour. Madelung potential indicates the cohesive strength around a site in the lattice of an ionic solid. Its magnitude is determined by the charge and distance of the site's nearest neighbours, so changes in coordination environment around the different surface sites would result in varying Madelung potentials. Generally, the lower the Madelung potential on an anion site, the more likely a vacancy is to form there, and the less negative the Madelung potential is on a cation site, the more likely electrons are to accumulate on it.

Here, we only consider the surfaces that appear on the Wulff shape, *i.e.* the (001), (101) and (211) facets, but similar behaviour can be expected on other surfaces as well. Madelung potentials for the bulk Y_2_Ti_2_O_5_S_2_ and the three surfaces were calculated using the Ewald summation method implemented in pymatgen and can be found in ESI Tables S1–S4.† To determine coordination environments, a maximum bond distance of 3 Å was used for Y, Ti and S and a lower threshold of 2.5 Å was used for O to avoid the spurious assignation of O–O bonding. Madelung potentials for Y were calculated for completeness but as Y states do not constitute the electronic band edges, Y sites are unlikely to be electronically active.

**Fig. 8:**
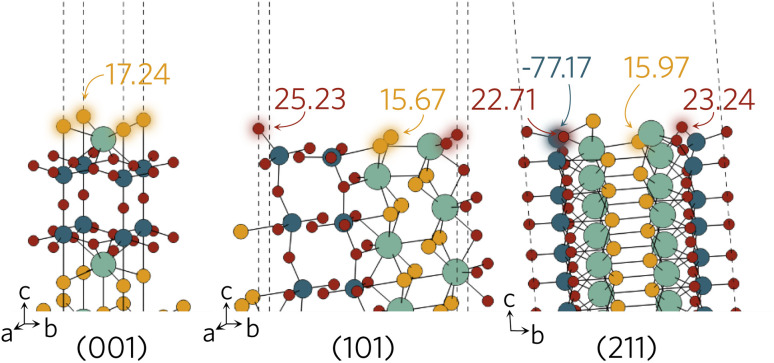
Surface active sites on (001), (101) and (211) surfaces shown with an aural glow with the corresponding Madelung potential of each site in V.

The Madelung potentials of Ti on the (001) and (101) surfaces are all within 0.5 V of the bulk-like potential. Because of the nature of the (211) surface cleavage, more changes to the coordination were seen; however, only two surface Ti see large changes to their electrostatic potential. The calculated potential of the under-coordinated Ti in the TiO_3_S motif in Fig. 8 is 3.2 V less negative than bulk-like; while the Madelung potential of the only fully-coordinated Ti on the surface is 4.2 V more negative.

Of the two distinct oxygen sites, O_ax_ is expected to be less likely to form a vacancy due to demonstrating at least 1 V higher potential compared to the O_eq_. There are no oxygens directly on the surface of the (001) slab and Madelung potentials reveal all have bulk-like values. This is of course not unexpected as the structure remains largely bulk-like after the geometry optimisation. A larger difference is seen on the (101) and (211) surfaces where the majority of under-coordinated surface oxygens experience a lower Madelung potential than those in the bulk-like region. Between the different surface sites, the greatest variation in potential was seen on the (211) surface, where the two-coordinate O_eq_ experience up to 6 V lower potential than the fully-coordinate counterparts (highlighted in Fig. 8). The opposite was found when analysing the sulphur Madelung potentials where the two-coordinate S in the TiO_3_S highlighted in Fig. 2b experiences the second highest surface Madelung potential due to the short Ti–S bond (2.32 Å).

The possible active sites based on Madelung potential analysis on the (001), (101) and (211) surfaces are summarised in Fig. 8. Clearly, the under-coordinated S and O_eq_ atoms with longer-than-average bond distances to neighbouring Y and Ti will form vacancies more readily than other surface anions. As the conduction band minimum is comprised of Ti d states, it follows that, when ionised, the charge is likely to accumulate on the under-coordinated Ti, reducing it to Ti^3+^. Obviously, we only considered what would happen to anion vacancies and where charge would accumulate on the cations. Intrinsic interstitials and antisites, as well as extrinsic dopants will also affect the photocatalytic activity and likely introduce other surface active sites, so further work is necessary to fully describe the Y_2_Ti_2_O_5_S_2_ surface defect chemistry.

### 2.5 Bulk optoelectronic properties

**Fig. 9:**
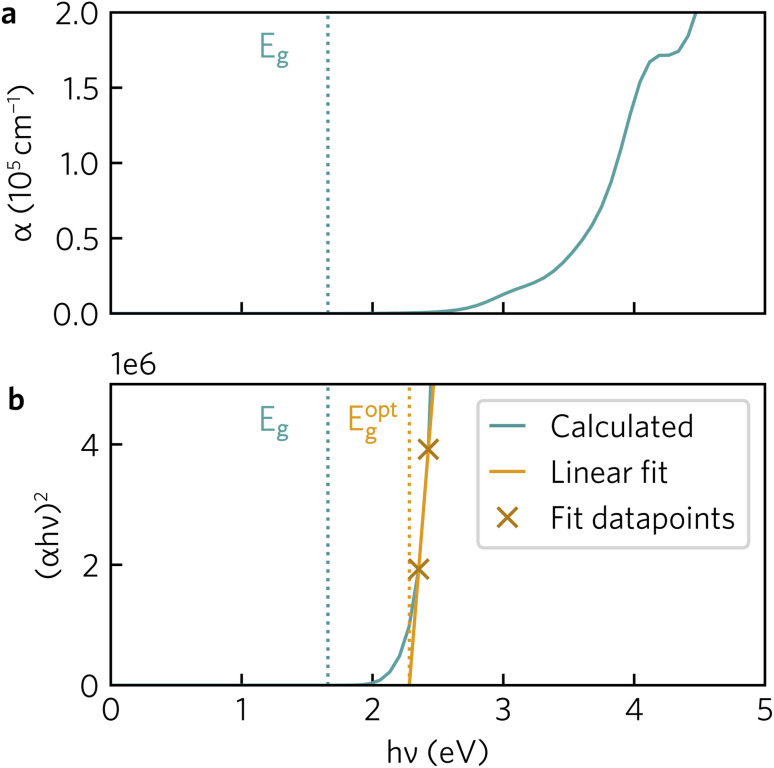
(a) Y_2_Ti_2_O_5_S_2_ HSE06-calculated absorption coefficients (*α*). (b) Tauc plot with line of best fit. Both plots include Gaussian smearing of 0.15 to improve readability. Fundamental (*E*_g_, 1.95 eV) and optical (*E*^opt^_g_, 2.28 eV) band gaps indicated in a dotted line.

The optical absorption spectrum calculated with the HSE06 functional in Fig. 9a shows a slow onset of absorption at 1.95 eV from the fundamental band gap, while the Tauc plot in Fig. 9b reveals a optical band gap of 2.28 eV. The irreducible representations analysis reveals the optical transition from the doubly-degenerate valence band maximum (VBM) to conduction band minimum (CBM) is parity allowed but is expected to be weak. From the topmost valence band, the band gap is 1.95 eV all the way along the *Γ*–*Z* direction where the band edges are perfectly flat. In theory an optical transition could occur, but *Z*–*Z* transitions are parity forbidden. Transitions at both *N* and *X* are parity allowed, but as the curvature in both valence and conduction bands is greater in *Γ*–*N* and *Γ*–*X* directions, the band gap rapidly increases until other combinations of transitions around band edges are lower in energy. The VBM to CBM+1 (0.30 eV above CBM) transition is symmetry forbidden; however, VBM−1 (0.32 eV below the VBM) to CBM is allowed. The latter VBM−1 to CBM transition is stronger and with an energy gap of 2.26 eV lines up well with the Tauc band gap.

**Fig. 10:**
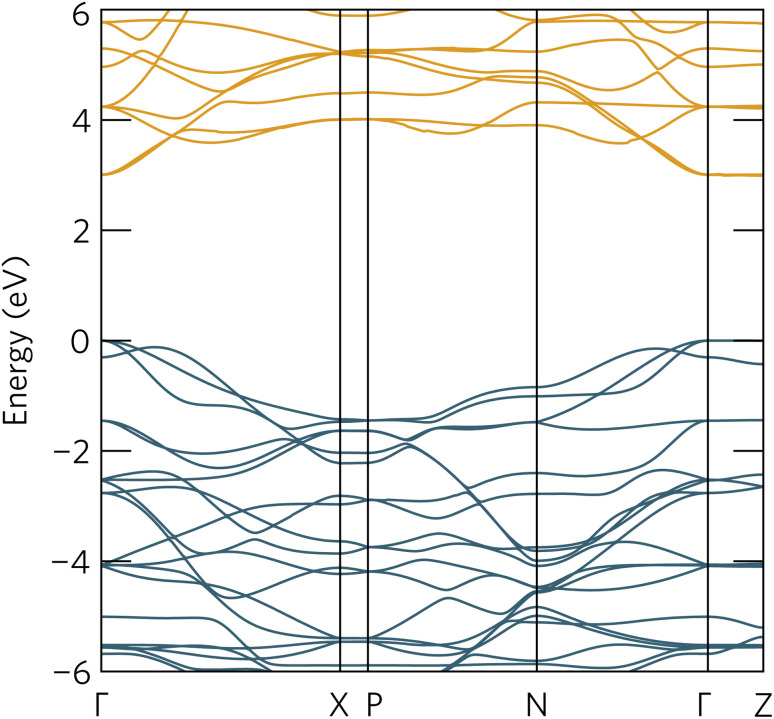
The quasiparticle band structure of Y_2_Ti_2_O_5_S_2_, calculated within QS*GŴ* method. The chosen *k*-path across the Brillouin Zone uses notation from Bradley and Cracknell.^52^ Valence bands are depicted in blue, conduction bands in orange, the valence band maximum is set to 0 eV, and the figure has been plotted using the sumo package.^53^

To further aid in the characterisation of Y_2_Ti_2_O_5_S_2_ as a photocatalyst, we explored its excitonic properties theoretically using many-body perturbation theory. First, we calculated the quasiparticle band structure using the QS*GW* and QS*GŴ* methods, to compare with our hybrid DFT calculations, using the HSE06 relaxed geometry. The band structures calculated with QS*GŴ* is depicted in Fig. 10, while the QS*GW* electronic structure can be found in the ESI Fig. S1.†

Initially, it is evident that the core features of the band structure, such as the dispersion along *Γ* to *X* and the correspondent localisation of states along *Γ* to *Z* are replicated in both quasiparticle methods here, as well as the HSE06 band structure. A minor change from HSE06 is the increased splitting of the degenerate Ti d conduction bands along *Γ* to *N*. More significantly, the quasiparticle gap is significantly greater −3.05 eV with QS*GW* compared to the fundamental HSE06 gap of 1.95 eV. QS*GW* is expected to systematically overestimate band gaps due to the omission of the vertex and incomplete screening effects within the RPA, typically only by 10% in s/p semiconductors, but the effect can be much larger in Mott–Hubbard insulators such as NiO.^32^ The addition of ladder diagrams, and thus additional screening due to the electron–hole interactions, to the screened Coulomb *W* in the QS*GŴ* method is expected to account for the majority of the latter effect – in Y_2_Ti_2_O_5_S_2_, it does reduce the gap, but only by 60 meV to 2.99 eV. This difference in band gap, of over 1 eV between the hybrid DFT and quasiparticle band structures, is considerable, though is similar to that seen in the caesium titanium halides, which similarly have an anionic-dominated valence band and Ti d conduction band.^54,55^ This difference will be revisited when considering the optical spectrum.

#### 2.5.1 Exciton binding energy

The exciton binding energy is the energy required to ionise an exciton from its lowest energy state to create a pair of dissociated free charge carriers. The exciton binding energy *E*_b_, for a Mott–Wannier-type exciton (where the exciton is delocalised) in an isotropic system can be approximated from:3
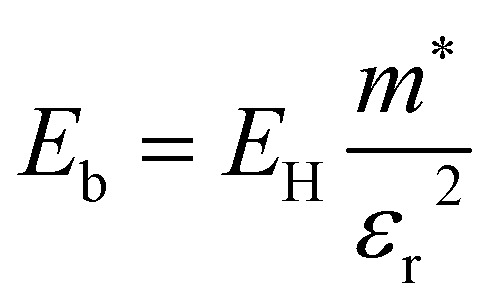
where *E*_H_ is the 1s orbital of hydrogen (−13.6 eV), *ε*_r_ is the dielectric constant of the material and m* is the reduced exciton mass, calculated from the electron and hole effective masses using 
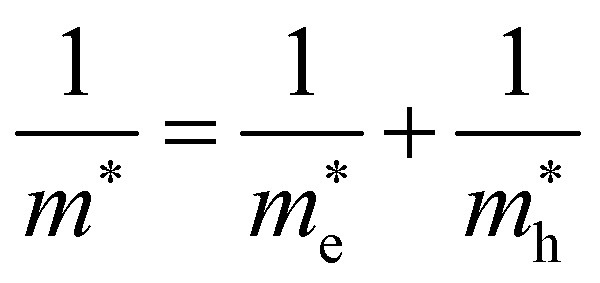
. The electronic structure of Y_2_Ti_2_O_5_S_2_ is highly anisotropic between the in-plane (*xy*) and out-of-plane (*z*) directions, so the simple hydrogenic model is not the most suitable. To account for the anisotropy, the eqn (3) is modified to describe a two-dimensional (2D) system:4
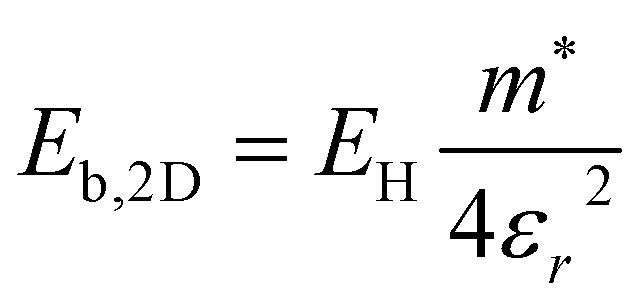
where the *ε* is the arithmetic mean of the static dielectric in the in-plane and out-of-plane directions, and the electron and hole effective masses used to calculate the reduced exciton mass m* are obtained from a harmonic mean of in-plane and the out-of-plane effective masses. For example, to calculate the effective electron mass the following formula was used:5
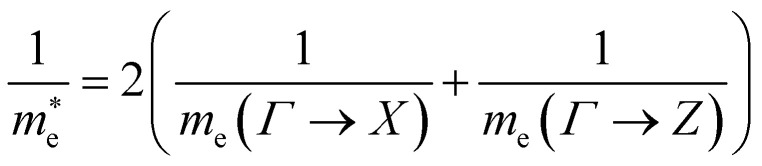


Using the electron and hole effective masses 0.82 *m*_e_ and 0.44 *m*_e_, respectively, and the calculated static dielectric constant of 30, the predicted exciton binding energy of Y_2_Ti_2_O_5_S_2_ is 1 meV. For photocatalysts for water splitting, the binding energy should be less than thermal energy (*k*_B_*T*), which is around 25 meV at room temperature. Evidently this crude estimate shows efficient separation of the charge carriers is possible at low temperatures.

**Fig. 11:**
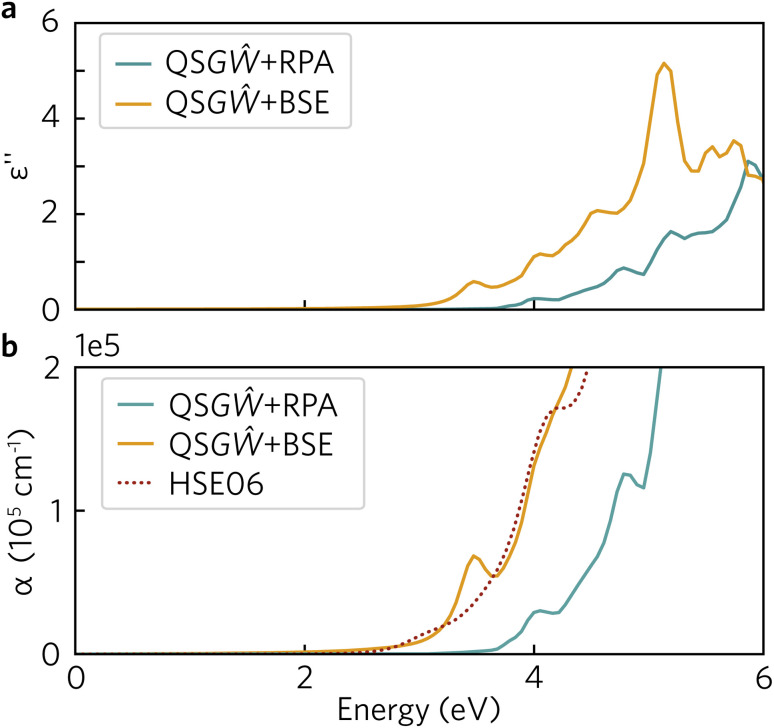
(a) Imaginary dielectric spectrum, calculated with QS*GŴ* + RPA and QS*GŴ* + BSE with an inherent smearing of 0.01 Ry; (b) Absorption coefficient, calculated with QS*GŴ* + RPA and QS*GŴ* + BSE and HSE06 – an additional Gaussian smearing of 0.02 eV was added to the QS*GŴ* spectra to match that of the HSE06 spectrum.

To fully understand the excitonic properties of Y_2_Ti_2_O_5_S_2_, however, we can explicitly calculate the electron–hole pair through the solution of the BSE. In single-shot methods such as *G*_0_*Ŵ*_0_ + BSE, all excitonic effects are combined and so by using QS*GŴ* for the underlying self-energy, any quasiparticle gap renormalisation as a result of electron–hole screening has already been accounted for. Thus, by comparing QS*GŴ* + RPA and QS*GŴ* + BSE spectra the exciton binding energy can be directly extracted. In Fig. 11, the imaginary dielectric spectrum calculated with QS*GŴ* + RPA and QS*GŴ* + BSE are depicted and the resultant absorption coefficients compared with HSE06.

In Fig. 11a, a large excitonic effect is clear, with a red-shift of the onset of the BSE spectrum of at least 0.5 eV. The major band-to-band peaks in the RPA are also replicated, with the transition at 5 eV being further augmented in intensity. While there is no strong excitonic peak below the 2.99 eV quasiparticle gap, the absorption is non-zero as a result of a series of weakly allowed excitonic transitions: 2.59, 2.72, 2.87, 2.91 and 2.94 eV. Each of these transitions is two-fold degenerate, with one component being forbidden (dark) and the other being very weakly allowed along *x*/*y* – this behaviour would partly align with the above finding in the HSE06 optical calculations that the fundamental gap at *Γ* is weakly allowed, and is parity forbidden across the flat bands between *Γ* and *Z*. The theoretical exciton binding energy is thus 0.4 eV, significantly higher than that predicted from the Wannier–Mott model – even when including the possibility of a 2D exciton due to the anisotropic electronic structure. It may be noted that although Y_2_Ti_2_O_5_S_2_ is predicted to have a high static dielectric constant, the high frequency component (seen in ESI Fig. S2†) is relatively small (∼4.6). Using the high-frequency dielectric constant alone, the 2D Wannier–Mott model would predict an order of magnitude increase in the exciton binding energy to 28 meV, though this would still be an order of magnitude lower than that predicted by the BSE calculations.

**Fig. 12:**
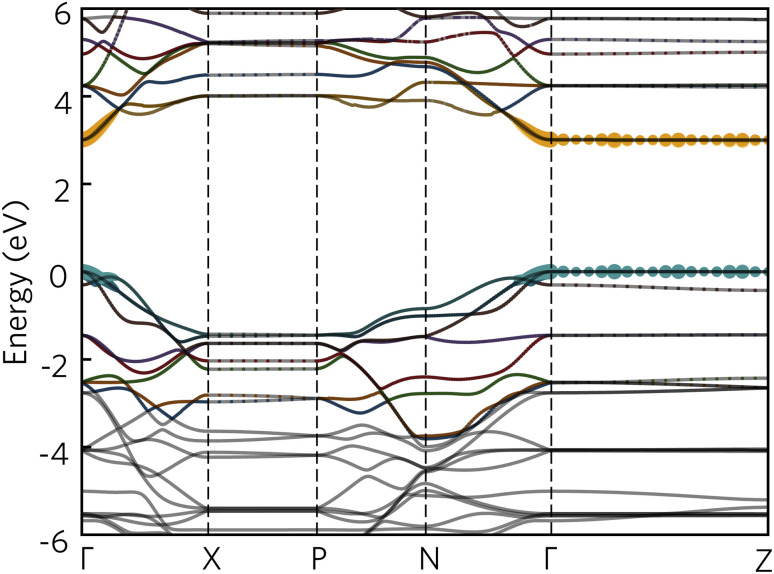
The quasiparticle band structure of Y_2_Ti_2_O_5_S_2_, calculated within QS*GŴ* method, with the magnitudes of individual state contributions to the first bright exciton (2.59 eV) projected onto the band structure. The chosen path across the Brillouin Zone uses notation from Bradley and Cracknell.^52^ Excitonic contributions from the valence bands are depicted in blue and conduction bands in orange, and was plotted with software included within the Questaal package.^30^

Further explanation may be provided by analysing the contributions to the sub-gap excitonic transitions, as shown by a projection of the contributions to the 2.59 eV excitonic wavefunction back onto the quasiparticle band structure, as shown in Fig. 12. The exciton demonstrates a hybrid behaviour between Wannier–Mott and Frenkel behaviour. As expected, contribution from the valence and conduction band arises from all *k*-points across *Γ* and *Z*, consistent with complete localisation in that direction and thus a 2D exciton; along the other directions from *Γ*, there are however still significant contributions from multiple *k*-points, despite the low effective masses of both electrons and holes in that direction. Most notably, in the valence band, there is additional contribution from the band below (‘VB−1’) where it crosses the highest energy band, particularly along *Γ* to *X*: this mixing may explain why the exciton transitions are further partially allowed as detailed above.

It is also possible to draw comparison to recent findings with G_0_*Ŵ*_0_ + BSE on anatase TiO_2_, which despite low effective masses, high dielectric behaviour and a 3D-connected crystal structure has recently predicted to have a strongly bound Frenkel-type exciton with 2D character.^56^ Baldini *et al.*^56^ rationalise the existence of such a localised exciton (with many states contributing to the transition) through the parallel curvature of valence and conduction bands in the electronic structure of anatase meaning electron and hole states have similar group velocities across a section of the Brillouin Zone. In Y_2_Ti_2_O_5_S_2_ not only are the valence and conduction bands parallel across *Γ* to *Z*, but ‘VB−1’ and the CBM are also parallel along *Γ* to *X* and along *Γ* to *N*, potentially leading to further localisation in other Cartesian directions as well, and a high exciton binding energy.

Nevertheless, with a binding energy of 0.4 eV, there is still a discrepancy between the predicted optical spectrum here and experimental photoluminescence emission that is observed centred around 2.0 eV.^57^ The QS*GŴ* method still does not include multiple effects that could further change the predicted optical behaviour of Y_2_Ti_2_O_5_S_2_, such as coupling of electrons or excitons to the lattice, or more complex phenomena such as bi-excitons or association of excitons with a defect centre. The former could be responsible for the larger portion of the observed discrepancy against experiment: a full study of exciton-phonon coupling is beyond the scope of this study, however, the approximate magnitude of the effect may be considered. The zero-point renormalisation of the gap of rutile TiO_2_ was recently calculated with *ab initio* methods to be 0.35 eV;^58^ were Y_2_Ti_2_O_5_S_2_ to demonstrate a reduction in quasiparticle gap by a similar magnitude, this would lead to the QS*GŴ* + BSE optical onset lying close to that observed experimentally. It is clear that further detailed study of the excitons in Y_2_Ti_2_O_5_S_2_ using both theoretical and experimental methods is necessary to properly characterise its optical properties and fully understand its capability as a photocatalyst.

A remaining question is then the suitability of hybrid DFT as a predictive tool, given that Y_2_Ti_2_O_5_S_2_ is predicted to be highly excitonic within many-body perturbation theory. In Fig. 11b, the absorption coefficients calculated using the three theoretical methods are compared within the relevant spectral region (up to the near-mid UV), and it is evident, that despite the absence of many-body effects within DFT, the HSE06 optical absorption matches very well to that of QS*GŴ* + BSE in both the slow initial onset followed and the strengthening above 3 eV, with the exception of the excitonically-enhanced peak in the latter above the quasiparticle gap. While this agreement may be fortuitous – though it may be noted that the functional form of HSE06 includes the effect of screening on the exchange interaction – it demonstrates that HSE06 can be reliable at generally predicting the optical behaviour and onset of Y_2_Ti_2_O_5_S_2_, and thus that the trends established using hybrid DFT are valid in comparison with higher-level theoretical methods.

## 3 Conclusions

In this work, we investigated the structures, energetics and electronic band alignments of clean Y_2_Ti_2_O_5_S_2_ non-polar surfaces with a maximum Miller index of 2 using DFT calculations, utilising both generalised gradient approximation and hybrid functionals. We outline the main relaxation patterns of the surfaces, which primarily occur when the TiO_5_S octahedra are cleaved along the O_ax_–Ti–S bond when surfaces are created.

Cleavage energies of the eleven studied Miller indices, calculated using the Fiorentini–Methfessel approach, all fall in the relatively wide 0.41–2.93 J m^−2^ range. The relaxed surface energies were up to 1 J m^−2^ lower compared to the cleavage energies, covering a slightly narrower 0.39–1.99 J m^−2^ range. The (001) surface was found to be the most stable with a surface energy of 0.39 J m^−2^. Only three surfaces, (001), (101) and (211), appeared on the Wulff construction representing the equilibrium form of Y_2_Ti_2_O_5_S_2_ at the thermodynamic limit. Overall, the general plate-like shape with the basal (001) plane agrees well with the reported experimental particle morphology.

The band alignment of the clean surfaces that appear on the Wulff shape agrees well with the experimentally observed spatial separation of hydrogen and oxygen evolution facets. The band alignment of the basal (001) surface suggests good overpotential for the oxygen evolution reaction, while the side (101) and (211) surfaces exhibit sufficient overpotential for hydrogen evolution. As this study was done assuming the surfaces are in vacuum, rather than in a aqueous medium, more future work would be needed to confirm the alignment in solution.

Potential surface active sites on the (001), (101) and (211) surfaces were identified using site Madelung potentials. Generally, sulphur and equatorial oxygen vacancies are expected to be the dominant surface active sites. On the (001) surface only the sulphur atoms experienced any variation in electrostatic potential, while both under-coordinated sulphur and equatorial oxygen sites are potential active site candidates on the (101). Due to the more complex cleavage direction on the (211) surface, larger differences in Madelung potentials between surface and bulk-like sites were observed. In addition to the sulphur and equatorial oxygen sites, the under-coordinated Ti could act as an active site, facilitating water redox by getting reduced to Ti^3+^.

The optical absorption of Y_2_Ti_2_O_5_S_2_, as calculated with both hybrid DFT and QS*GŴ* + BSE methods demonstrates a slow onset due to forbidden transitions at the fundamental gap, with stronger absorption at energies above 3 eV. Furthermore, QS*GŴ* + BSE calculations that explicitly include electron–hole interaction, predict an exciton binding energy of 0.4 eV, indicating at least partial Frenkel behaviour. A tightly bound 2D exciton is predicted as a result of not only the localisation of electrons and holes in the crystallographic *z* direction, but also additional mixing of multiple valence bands into the excitonic wavefunction. These results indicate that to fully understand the promise of Y_2_Ti_2_O_5_S_2_ as a photocatalyst, its optical and excitonic behaviour needs further consideration.

## Author contributions

KB: investigation, methodology, data curation, formal analysis, visualisation, writing – original draft. CNS: investigation, formal analysis, writing – original draft. DOS: funding acquisition, supervision, writing – review & editing.

## Conflicts of interest

There are no conflicts to declare.

## Supplementary Material

TA-011-D3TA02801A-s001
