# Supplementary material for: Understanding the electronic structure of Y2Ti2O5S2 for green hydrogen production: a hybrid-DFT and GW study
Source: J Mater Chem A Mater. 2023 Jul 20;11(31):16776–87. doi: 10.1039/d3ta02801a (PMC10408711; doi:10.1039/d3ta02801a)
Supplement: TA-011-D3TA02801A-s001 [file TA-011-D3TA02801A-s001.pdf]

Electronic Supplementary Material (ESI) for Journal of Materials Chemistry A.
This journal is © The Royal Society of Chemistry 2023

Supporting Information for "Understanding the Electronic Structure of $Y_2Ti_2O_5S_2$ for Green Hydrogen Production: A Hybrid-DFT and GW Study"

Katarina Brlec, Christopher N. Savory, David O. Scanlon

Department of Chemistry and Thomas Young Centre, University College London, London, United Kingdom

Table S1: Local bonding environments and average Madelung potentials for all Y sites in bulk $Y_2Ti_2O_5S_2$ and on the (001), (101) and (211) surfaces

Structure	Site	Coordination number		Average distance (Å)		Average Madelung potential (V)
		Y–S	Y–O	Y–S	Y–O	
Bulk		5	4	2.83	2.42	-42.25
(001)	bulk-like	5	4	2.83	2.42	-36.60
	surface	4	4	2.79	2.42	-36.58
(101)	bulk-like	5	4	2.83	2.42	-39.81
	surface	4	4	2.79	2.39	-39.37
	surface	3	3	2.77	2.28	-38.84
(211)	bulk-like	5	4	2.83	2.42	-38.23
	surface	2	4	2.84	2.34	-40.81
	surface	2	2	2.56	2.22	-37.08
	surface	4	4	2.80	2.41	-37.68
	surface	4	3	2.75	2.37	-39.69

Table S2: Local bonding environments and average Madelung potentials for all Ti sites in bulk $Y_2Ti_2O_5S_2$ and on the (001), (101) and (211) surfaces

Structure	Site	Coordination number		Average distance (Å)		Average Madelung potential (V)
		Ti–S	Ti–O	Ti–S	Ti–O	
Bulk		1	5	2.89	1.91	-86.05
(001)	surface	1	5	2.92	1.91	-78.22
	bulk-like	1	5	2.89	1.91	-78.56
(101)	bulk-like	1	5	2.89	1.91	-82.94
	surface	1	4	2.82	1.87	-82.25
	surface	0	5	–	1.93	-82.46
	sub-surface	1	5	2.72	1.90	-83.66
	sub-surface	1	5	2.96	1.91	-82.24
(211)	bulk-like	1	5	2.89	1.91	-80.36
	surface	1	3	2.32	1.79	-77.17
	surface	0	5	–	1.94	-80.54
	surface	0	4	–	1.84	-80.81
	sub-surface	1	5	2.89	1.92	-79.42
	surface	1	5	2.94	1.96	-84.58

Table S3: Local bonding environments and average Madelung potentials for all S sites in bulk $Y_2Ti_2O_5S_2$ and on the (001), (101) and (211) surfaces

Structure	Site	Coordination number		Average distance (Å)		Average Madelung potential (V)
		Y–S	Ti–S	Y–S	Ti–S	
Bulk		5	1	2.83	2.89	15.90
(001)	surface	4	1	2.83	2.90	17.24
	bulk-like	5	1	2.83	2.89	19.65
(101)	surface	3	1	2.79	2.83	15.67
	surface	5	0	2.76	3.05	18.56
	bulk-like	5	1	2.83	2.91	17.49
(211)	surface	3	1	2.79	2.94	15.97
	surface	1	1	2.78	2.32	17.10
	surface	4	0	2.78	3.29	16.85
	surface	4	0	2.74	3.09	18.96
	bulk-like	5	1	2.83	2.91	18.45

Table S4: Local bonding environments and average Madelung potentials for all O sites in bulk $Y_2Ti_2O_5S_2$ and on the (001), (101) and (211) surfaces

Structure	Site	Coordination number		Average distance (Å)		Average Madelung potential (V)
		Y–O	Ti–O	Y–O	Ti–O	
Bulk	axial	0	2	–	1.79	26.83
	equatorial	2	2	2.42	1.94	27.34
(001)	bulk-like axial	0	2	–	1.79	30.57
	bulk-like equatorial	2	2	2.42	1.94	31.17
(101)	bulk-like equatorial	2	2	2.42	1.94	28.96
	bulk-like axial	0	2	–	1.79	28.35
	surface equatorial	2	1	2.27	1.90	25.23
	surface equatorial	0	2	–	1.91	26.77
	surface axial	0	2	–	1.82	27.49
(211)	bulk-like axial	0	2	–	1.79	29.76
	bulk-like equatorial	2	2	2.42	1.94	30.14
	surface axial	0	2	–	1.86	28.21
	surface axial	0	2	–	1.82	29.35
	surface equatorial	1	1	2.23	1.70	23.25
	surface equatorial	1	1	2.21	1.88	22.72
	surface equatorial	2	1	2.24	1.88	25.11
	surface equatorial	1	2	2.23	2.02	29.78
	surface equatorial	2	2	2.39	1.93	29.62
	surface equatorial	1	2	2.45	1.91	31.48

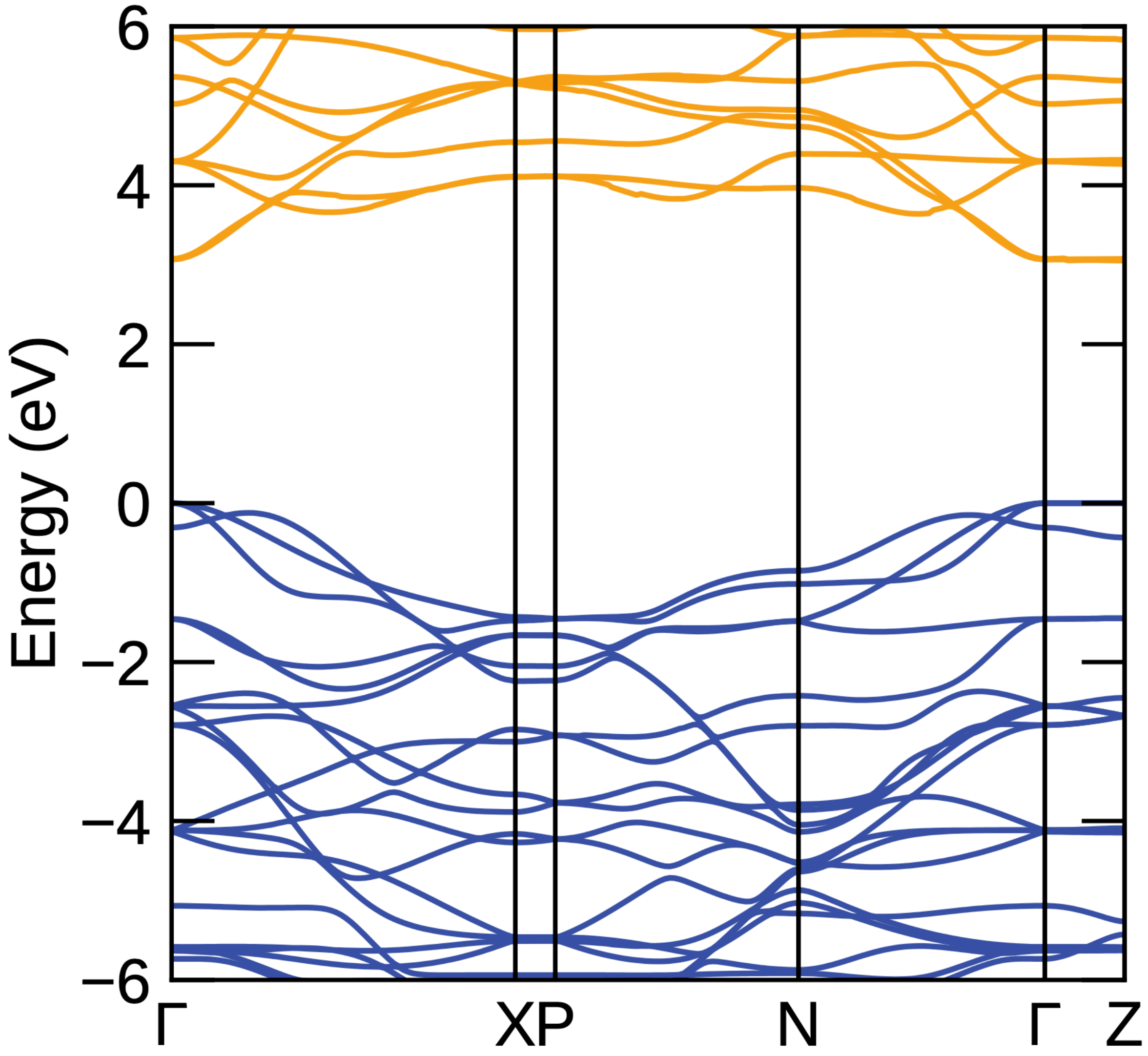

Figure S1: QS*GW* band structure, plotted with sumo[1] along the Bradley-Cracknell[2] k-point path.

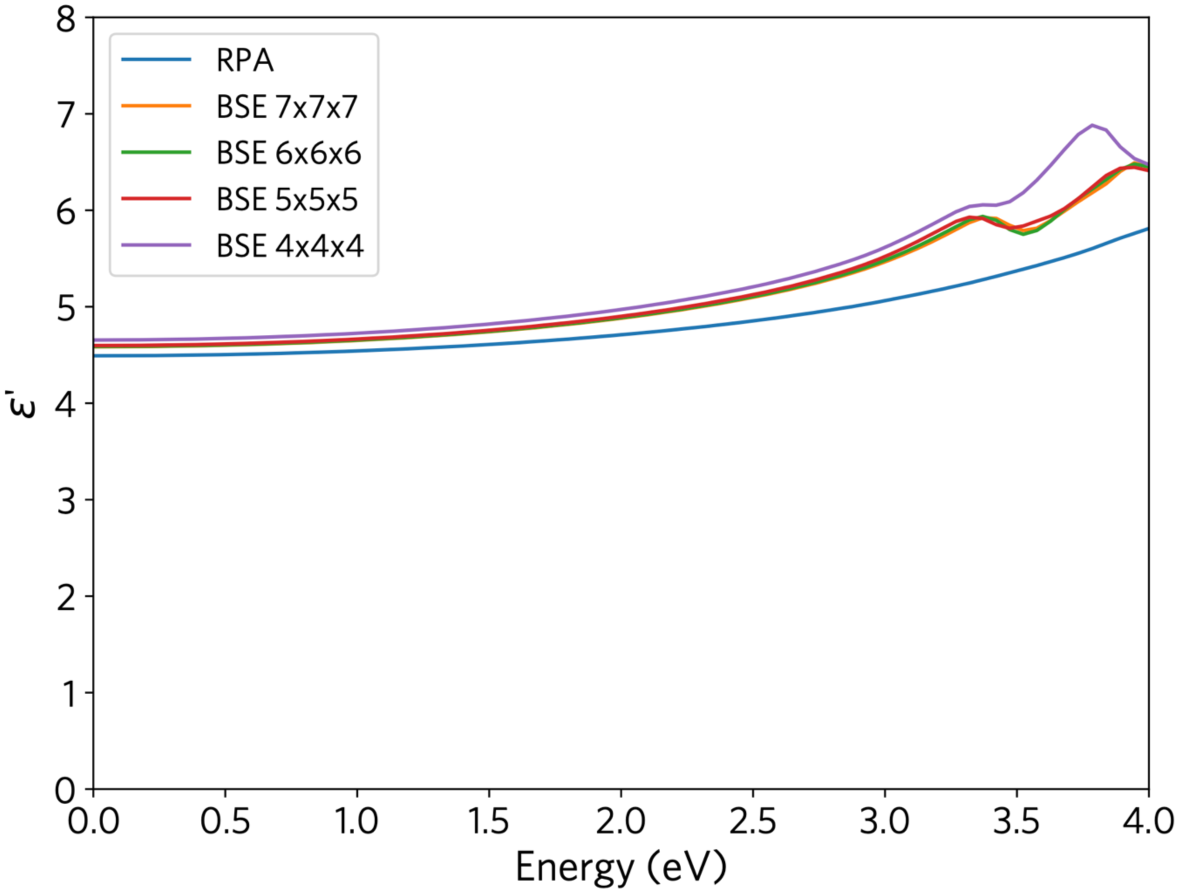

Figure S2: Convergence of the real part of the high-frequency dielectric constant from QS$G\hat{W}$ solved with the Bethe-Salpeter Equation (BSE) with respect to the k-point mesh. QS$G\hat{W}$ within the random phase approximation (RPA) plotted for comparison.

Additional information, containing input and output files for all surface calculations and details of analysis and plotting, including the specific scripts used are available on Zenodo: 10.5281/zenodo.7924906

References

[1] A. M. Ganose, A. J. Jackson and D. O. Scanlon, *J. Open Source Softw.,* 2018, 3, 717.
[2] C. J. Bradley and A. P. Cracknell*, The Mathematical Theory of Symmetry in Solids: Representation Theory for Point Groups and Space Groups*, Clarendon Press, Oxford, 1972
